# Palliative care in patients with glioblastoma: A systematic review

**DOI:** 10.3892/mi.2025.245

**Published:** 2025-05-28

**Authors:** Ahmed M. Al-Ghaithi, Sara Al-Barkhati, Al Munqith Al Abri, Doaa Al Husaini, Abdallah Al-Hajri, Tariq Al-Saadi

**Affiliations:** 1 Department Neurosurgery, College of Medicine and Health Sciences, Sultan Qaboos University, Muscat 132, Sultanate of Oman; 2 Department of Ophthalmology, Sultan Qaboos University Hospital, Muscat 132, Sultanate of Oman; 3 Department of Neurosurgery, Khoula Hospital, Muscat 113, Sultanate of Oman; 4 Department of Neurosurgery-Cedars-Sinai Medical Centre, Los Angeles, CA 90048, USA

**Keywords:** glioblastoma, grade 4 glioma, palliative care, conservative, non-surgical, comfort care, management, palliative radiotherapy, palliative chemotherapy, end-of-life

## Abstract

The present study aimed to systematically review and analyze the available literature on patients with glioblastoma (GBM) receiving palliative care. A systemic literature review was thus conducted searching for patients with GBM receiving palliative care using the following databases: PubMed, Scopus, Wiley and Web of Science. All articles relevant to the review were included, covering all age groups and types of palliative care used in all settings, and there was no time limit. A total of 234 studies were obtained from the search that matched the objectives of the review. A total of 19 articles were included, ranging from 1998 to 2022, with a total number of 7,392 patients. Supportive treatment was one approach to palliative and end-of-life care in patients with GBM; other approaches included chemotherapy, surgery and radiotherapy. The location of the mortality of patients with GBM differed between hospitals and health institutes, homes and hospice care. Out of 19 studies included, 10 of these reported hospitals to be the place of mortality. Patients with GBM have a poor prognosis, with a poor survival rate, even with the optimal treatment available. Moreover, multiple signs and symptoms can burden the end-of-life of patients and their families. Palliative care in these patients aims to relieve the burden of end-of-life care and improve the quality of life of the patients and their families.

## Introduction

Primary brain tumors comprise a heterogeneous group of neoplasms, with different outcomes, with patients requiring different management strategies. These tumors can range from pilocytic astrocytomas, a very uncommon, non-invasive curable tumor, to glioblastoma (GBM), which is associated with more invasive and aggressive behaviors (1).

GBM is the most common primary brain tumor among adults. It is associated with a median survival rate of 16-21 months and a 10-year survival of <1% (1-5). GBM accounts for 45.6% of all primary brain malignancies. The incidence rate of GBM is 3.19 among 100,000 individuals from different age groups with a median age of 64 years; however, it can occur at any age (6).

In general, GBM is associated with a very poor prognosis. However, several parameters associated with improved outcomes include an age <50 years, a non-eloquent tumor location, a Karnofsky performance status (KPS) score ≥70 and the maximal extent of tumor resection (7,8).

The treatment of patients with GBM includes maximal surgical resection with adjuvant radiotherapy. The inclusion of nitrosoureas has exhibited benefits in addition to the standard treatment; however, this has only been demonstrated in multivariant and randomized comparison studies (7,9). The use of adjuvant temozolomide was previously investigated in a randomized phase III, EORTCNCIC trials along with standard surgical resection and radiotherapy; its use was found to be associated with an improved overall survival rate of 14.6 compared to 12.1 months with standard treatment with radiotherapy (10,11). Temozolomide was approved in 2005, and since then, it has been the standard chemotherapeutic treatment for GBM for six 6 cycles following radiotherapy (12).

GBMs have a high recurrence rate even in cases in which they have been discovered at an early stage and treated completely. The median recurrence time is 9.5 months, with an overall survival rate of 30 months (13). The treatment of recurrent or progressive GBMs can include supportive care, as decided by the treating physician. On the other hand, tumor-specific multidisciplinary boards are another approach for treating patients with GBM; the use of these has been shown to be associated with a 12-month survival rate of 32.5% compared to 11.3% in the group with supportive treatment (14).

A previous meta-analysis investigated palliative care intervention in adults with terminal illnesses and diseases, including oncology. The quality of life (QOL) of patients was assessed in 24 studies, including 4,576 patients; 12 (50%) studies evaluated the association between QOL and palliative care intervention and reported a statistically significant improvement in QOL and symptoms burden (15).

When assessing the end-of-life in a patient with GBM, a decreased level of consciousness, a change in mental status, fever, seizures and dysphagia have been shown to have the most marked clinical burden as the disease progresses. Moreover, this provides the basis for care in these terminal care cases to include anticonvulsants, steroids and gastric protection, such as non-steroidal anti-inflammatory drugs (16,17).

Other modalities of palliative care are short-course radiotherapy, which has been shown to be beneficial in patients with a KPS score <50, along with the optimal supportive and palliative care, including the use of corticosteroids (18). The use of mifepristone, a progesterone receptor antagonist, has also been suggested for palliative care therapy in patients with advanced-stage brain tumors, including GBM, as it exhibits good penetration through the blood-brain barrier (19).

The American Society of Clinical Oncology (ASCO) Clinical Practice Guidelines recommend the addition of palliative care in patients with advanced-stage cancer (20). Specifically, patients with GBM suffer from progressive neurological diseases that affect their QOL along with their decision-making capacity; of note, ~50% of patients with primary malignant brain tumors have compromised medical decision-making at the time of diagnosis due to cognitive impairment, behavioral changes and poor communication abilities (21). Therefore, advanced care planning (ACP) has evolved to facilitate the communication of goals and preferences regarding future medical care, and it is considered crucial in patients with GBM. It not only includes the treatment design and a proxy decision maker, but also extends to involve open communications between the patient, proxy, decision-makers and care providers to discuss the preferences for future medical care, including palliative care options (22).

ACP can be utilized to improve the quality of communications between patients and healthcare providers and may reduce unwanted interventions and admissions. In addition, it enhances the use of palliative care, which increases the satisfaction and QOL of both patients and relatives (22,23).

Therefore, the present study aimed to systematically review and analyze the available literature on patients with GBM receiving palliative care.

## Data and methods

### Literature search strategy

The present study aimed to systematically review and analyze the available literature on patients with GBM receiving palliative care. The PubMed, Scopus, Wiley and Web of Science databases were searched by three authors (AMAG, SAB and AMAA) to gather the available literature using the following key words: ‘Glioblastoma’, ‘GBM’, ‘Grade 4 glioma’, ‘Palliative care’, ‘Conservative’, ‘Non-surgical’, ‘Comfort care’, ‘Management’, ‘Palliative Radiotherapy’, ‘Palliative Chemotherapy’ and ‘End of life’.

### Study selection, inclusion and exclusion criteria

Studies were selected using a systematic review following the Preferred Reporting Items for Systematic Reviews and Meta-Analysis (PRISMA) guidelines. All articles relevant to the topic of the review were included, covering patients of all age groups, and all types of palliative care used, in all settings and there was no time limit; however, articles that were not published in the English language were excluded from the systematic review.

### Data extraction and analysis

After applying the inclusion and exclusion criteria, AMAG, SAB and AMAA screened the titles and abstracts of possible eligible studies. Moreover, the three authors examined the key features from the eligible studies, extracting the aims, treatments and palliative care applied, as well as the outcomes, place of mortality (either at a health institute or at home), and the recommendation from the authors of that study. In addition, the three authors examined the year of publication, and the country where the study was conducted.

## Results

### Study selection, including and exclusion criteria

**Figure 1:**
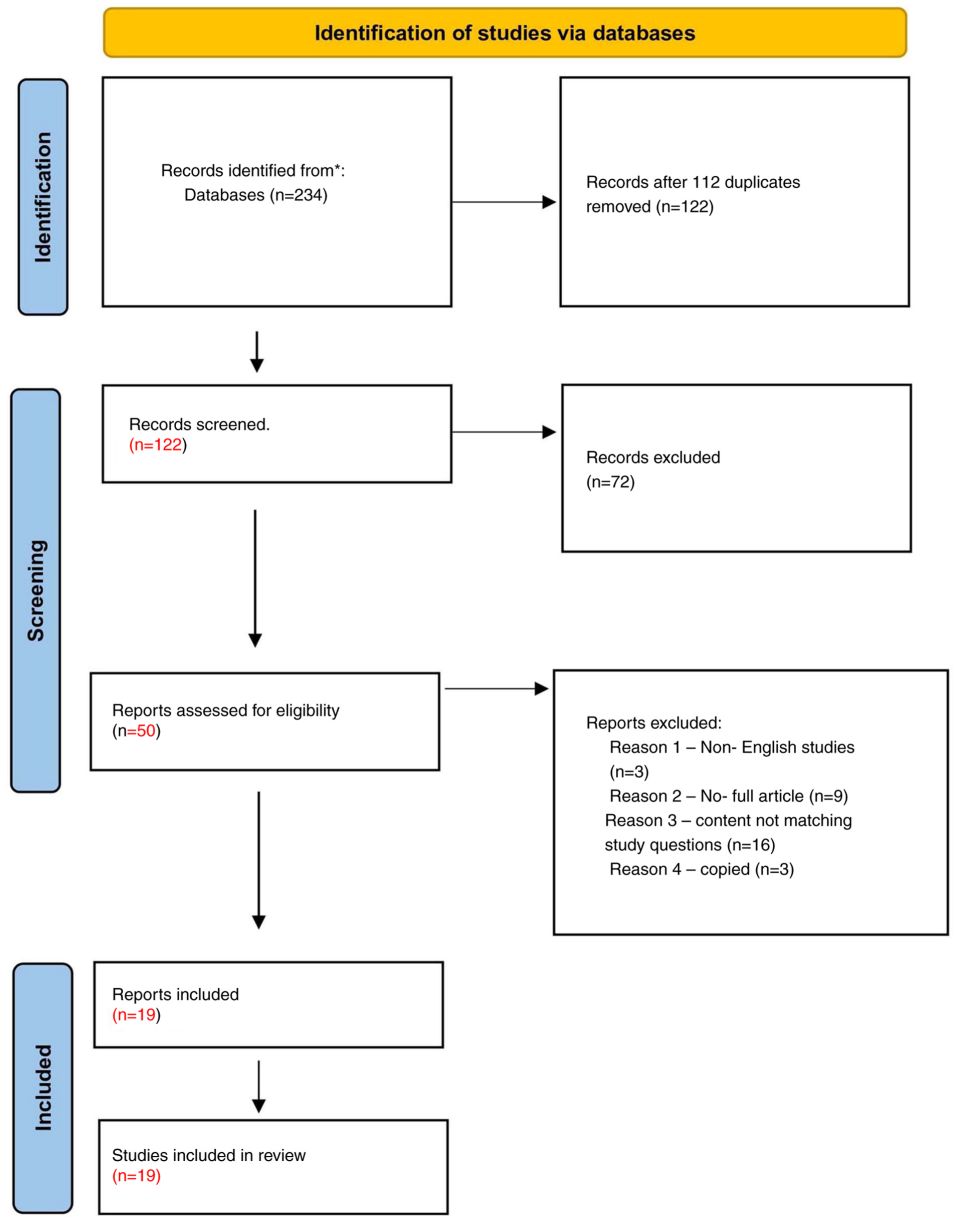
Preferred Reporting Items for Systematic Review and Meta-Analysis (PRISMA) flow chart. A total of 234 studies were obtained; 122 studies removed as they were duplicates. After excluding non-English studies, incomplete articles, 19 articles were included.

In the present systematic review, the three authors were allocated to investigate four databases, obtaining a total of 234 studies that matched the objectives of the review. Moreover, 112 studies were excluded as they were duplicates. After reviewing these articles, 72 studies were removed as they did not match the aim of the review. After applying the inclusion and exclusion criteria, a total of 50 articles were included; however, of these, three articles were excluded as they were non-English studies, the full article could not be accessed in nine articles and 16 articles were not relevant to the study question. In addition, three studies were identified as copies or duplicates, having been retrieved from the searches conducted independently by the three different authors and were removed. Eventually, a total of 19 articles were included in the present systematic review (Fig. 1).

### Quality assessment and geographical distribution

**Figure 2:**
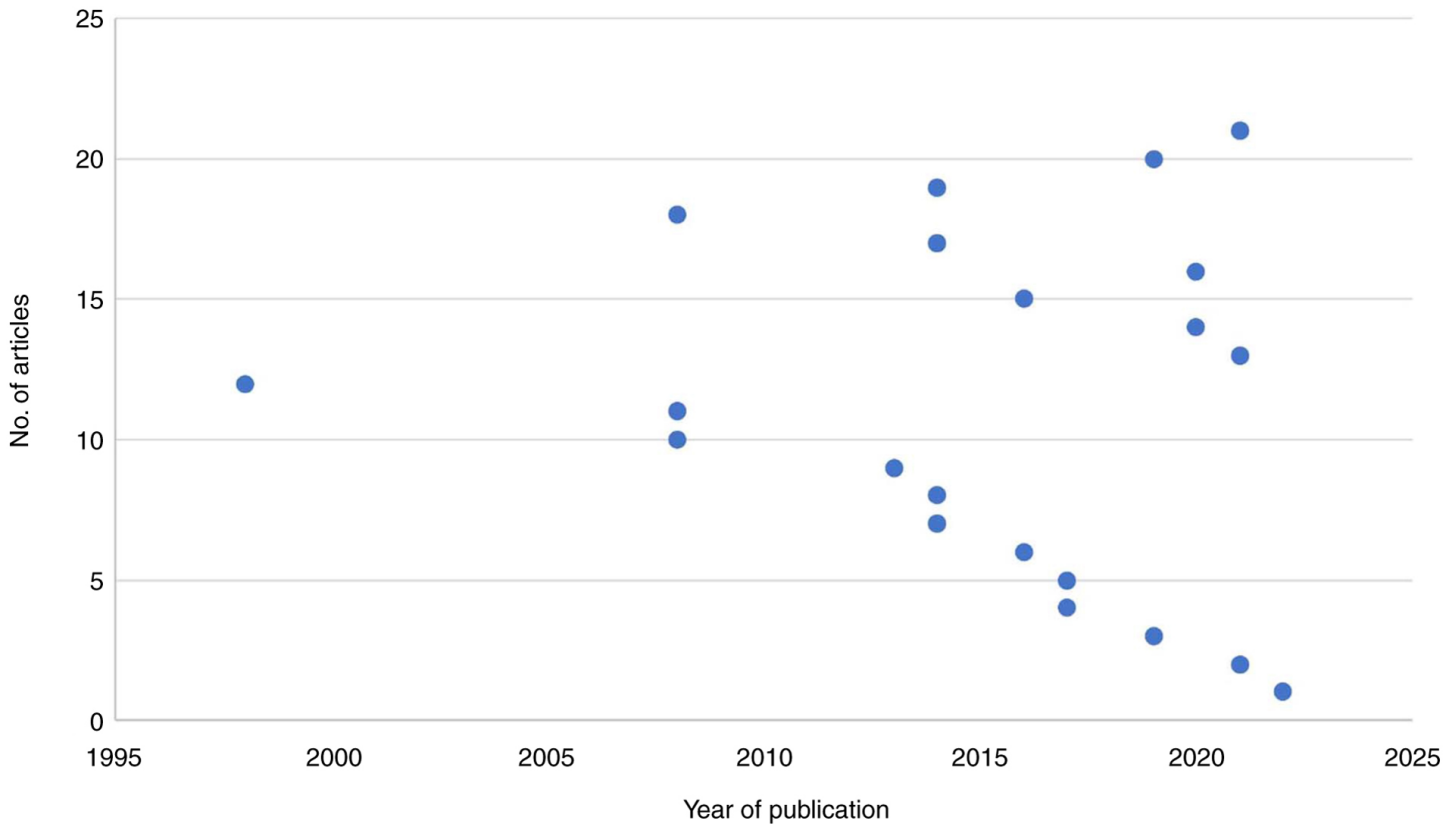
The publication year of the included articles in the present study. Over a wide range of years (1998-2022), the highest number of publications (n=4) were in 2014. Additionally, the least number of publications were found in 1998, 2013, 2016 and 2022.

In the present systematic review, 19 articles were included, with publication years ranging between 1998 and 2022, with a total of 7,392 patients (Fig. 2). Of note, two of these articles were case reports (19,24). The majority of the included articles were retrospective analyses (10 articles out of 19 articles) (14,18,25-32). A total of four articles were prospective studies (16,33-35), two articles were systematic reviews (17,36) and one article was a randomized clinical trial (37) (Table I).

The present systematic review included articles conducted in a variety of countries (Table II); the majority of articles were from the USA (17,19,25,28,29,35,36), and the remaining articles were from Germany (14,18,33,37), Austria (16,32), Australia (26,30), Poland (31), Italy (34), Ireland (27), and one study published by authors from different nationalities (24).

### Palliative care

From each included study, different aspects were evaluated, including the primary treatment administered if applicable, the palliative care treatment introduced, whether the study examined inpatients, outpatients, or both, and the outcomes derived from each intervention. The median survival rate was evaluated, and the recommendation was provided by the authors. In the included studies, different palliative care therapies were used as adjuvants with the primary treatment, targeting various aspects of palliative care (Table III).

Supportive treatment was one approach to palliative and end-of-life care in patients with GBM, as Pompili *et al* (34) aimed to identify home palliative care and end-of-life issues in patients with GBM. They found that midazolam was necessary in 11% of cases to achieve good control of symptoms, such as delirium, agitation and refractory seizures. In addition, phenobarbital was the drug of choice for severe seizures, which occurred in 30% of cases (34).

Moreover, the use of phenobarbital was assessed by Senderovich *et al* (24), in an end-of-life setting; its use was found to reduce complications associated with end-of-life care and improve the quality of remaining life (24).

Kuchinad *et al* (29) conducted a retrospective analysis on the management of patients with GBM, focusing on end-of-life care practices at an academic center. Their study primarily evaluated the use of chemotherapy as the main treatment approach for patients with GBM, without exploring palliative interventions. By comparing service utilization to national quality care guidelines, the researchers identified gaps in documentation related to palliative care and end-of-life planning. Their findings suggested that improving these aspects could enhance the overall quality of care provided to patients with GBM (29).

In patients receiving the full course of treatment, including surgery, chemotherapy and radiotherapy, multiple palliative care interventions were used to improve the quality of life of these patients. Among such studies, Oberndorfer *et al* (32) focused on symptomatic management, including antiepileptic drugs (AEDs), steroids and analgesia, physiotherapy, and occupational therapy in end-of-life patients. They classified the end-of-life into phases, from phase 1 to 3. These interventions were associated with symptomatic improvement in end-of-life patients, particularly when introduced via the non-oral route, given that the majority of patients developed dysphagia at this stage (32).

Lin *et al* (26) investigated steroids, AEDs, benzodiazepines and allied health involvement. They found that early palliative care resulted in a significant improvement in pain, somnolence, symptoms and distress score; they recommended the initiation of palliative care not only with medication treatment, but also with rehabilitation, along with psychosocial support (26).

Stavrinou *et al* (14) compared supportive care and second-line, tumor-focused treatment at first progression in two different groups. They found that second-line treatment, which is tumor-focused, is more effective in terms of outcomes and in terms of overall survival (14).

Apart from supportive care, Ziobro *et al* (31) examined the effects of palliative treatment with temozolomide in patients with high-grade gliomas. They found this treatment to be beneficial in 49% of patients in the study group (31).

Overall, standardizing guidelines for end-of-life care in patients with GBM was suggested by Thier *et al* (16), when they studied the symptoms and signs in the last 10 days prior to mortality, and how these could affect the health and care of patients (16).

In studies using radiotherapy and surgery as the primary treatment for GBM, Witteler *et al* (18) used radiotherapy as a palliative care treatment and found that it increased the survival rate, and that it was a reasonable option for patients with a limited prognosis. On the other hand, Reimer *et al* (33) used laser-induced thermotherapy (LITT) and found that interventional MRI controlled LITT and that it provided potential treatment benefits; MRI provides excellent topographic accuracy due to its capability for soft tissue contrast with high specific resolution and functional aspects (33).

### Location of mortality

The location of mortality of patients with GBM differs between hospitals and health institutes, homes and hospice care. Out of the 19 studies included in the present systematic review, 10 studies reported hospitals as the place of mortality (Table IV) (16,17,24,26,28-30,32,34,36).

Wu *et al* (36) performed a systematic review of palliative care service utilization and advance care planning. They demonstrated that the location of mortality was mentioned in only six out of the 16 studies included, and they similarly found that mortality in health care institutes was the most common compared to other locations, reaching up to 78% (36).

Mortality at home was reported in seven studies, with the numbers of patients varying from 12 to 53% (17,19,28-30,34,36). On the other hand, hospice care was the least mentioned among the included studies as the site of mortality (28,29,34,38). Wu *et al* (36) found that the mortality rate in this setting ranged from 12to 64%. However, Sundararajan *et al* (30) found that this rate was 49%.

## Discussion

The present study reviewed and systematically analyzed the available literature on patients with GBM receiving palliative care. GBM is considered to be the most common type of brain tumor in adults. It accounts for 45.6% of all brain tumors (1-5). It is generally associated with a very poor prognosis, as well as with a high recurrence rate (7,8,13). The treatment of patients with GBM includes maximal surgical resection with adjuvant radiotherapy (7,9).

Other modalities of treatment include supportive care as decided upon by the treating physician; however, other researchers advocate for tumor-specific multidisciplinary approaches to plan the treatment (14). Palliative care is currently recommended by the ASCO Clinical Practice Guidelines to be considered when treating patients with GBM (20).

A variety of studies investigating the management and palliative care of patients with GBM have been published. The present systematic review included publications over a wide range of years, from 1998 to 2022. It was found that 2014 accounted for the highest number of publications, which included four publications, followed by 2021 (Fig. 2). However, Wu *et al* (36) demonstrated that 2014, 2017 and 2018 were the years with the highest number of publications. Moreover, Ironside *et al* (38) found that 2012 was the year with the highest number of publications.

### Mean survival age

As GBM is a disease that is associated with a poor prognosis, improving the QOL and prolonging the life expectancy of patients is the main aim of palliative care, not only for patients but also for their families (39). The total number of patients who were diagnosed with grade 4 GBM between 2004 and 2017 and received palliative care was 2,803(40). Compared to the results of the present study, the total number of patients who received palliative care was 1,630, and this was expected, as some of the selected articles did not mention the exact number of patients who received palliative care from GBM. In terms of the ability to carry out daily activities, the studies have a KPS >50% with variable modalities of palliative care. Specifically, patients who received radiotherapy had a KPS score >60%, which is consistent in comparison with other studies that reported patients who received radiotherapy had a KPS score >60% (38).

### Palliative care in GBM

In the present systematic review, the median survival rate reached 14 months, which was consistent with a recently published retrospective study by Mohammed *et al* (41). Providing the optimal treatment when dealing with patients who require palliative care is critical; it is highly recommended to establish a specific and well-structured palliative care guideline for patients with GBM (16,32). Moreover, further research is warranted to define appropriate symptom management for those patients, which will be also helpful in the process of establishing GBM palliative care guidelines (17,34,42). Making these guidelines universal will ensure the right of patients to receive all and appropriate methods of palliative care and participation from multiple specialties recommended to target and deliver the optimal options for the patient (34).

### Location of mortality

The location of mortality for patients with GBM varies across hospitals, homes and hospice care. Out of the 19 studies included in the present systematic review, 10 of these reported the hospital as the place of mortality (16,17,24,26,28-30,32,34,36). This may be explained by the late hospitalization, particularly in intensive care, which has resulted in mortality in acute hospital care (43). Wu *et al* (36) performed a systematic review of palliative care service utilization and advance care planning, and demonstrated that the location of mortality was mentioned in only six out of 16 studies included; they similarly found that mortality in health care institutes was the most common compared to other places, reaching up to 78% (36).

However, Sundararajan *et al* (30) performed a retrospective study and found that 25% of the patients died in an acute hospital bed. Thus, the majority of the patients prefer to die at home, as shown by Barbaro *et al* (43). Moreover, home deaths were reported to range from 12 to 53.1% (17,19,28-30,34,36), which is less than the number reported by Wu *et al* (36) in their review.

As regards hospice care, the analysis revealed this to be the least common site of mortality (17,29,30,34). Wu *et al* (36) found that the mortality rate in this setting ranged from 12 to 64%. However, Sundararajan *et al* (30) found that this rate to be 49%.

### Limitations and future implications

The present systematic review was not without any limitations. Moreover, with such a prolonged study period, limited studies were found focusing on palliative care at the end-of-life for patients of GBM. In addition, end-of-life care can be challenging, and subjective measures will be limited to assess the needs of patients, and the outcomes of such an intervention, which will limit the study outcome.

In conclusion, patients with GBM have a poor prognosis and a poor survival rate, even with the optimal treatment available. Moreover, multiple signs and symptoms can have a tremendous burden on the end of life of patients and their families. Palliative care in these patients aims to relieve the burden of end-of-life care and improve the quality of life for them and their families. Different palliative care options were studied that led to the effective relief of patient symptoms, including symptomatic treatment, the involvement of other services, such as physiotherapy and some medications targeting each symptom and radiotherapy. Overall, the early planning and involvement of these services are critical and have a notable impact on the end-of-life of patients.

## Figures and Tables

**Table I:** Quality assessment of the included studies.

Article no.	Authors, year of publication	Type of study	Level of evidence	Sample	(Refs.)
1	Senderovich *et al*, 2022	Case report	4	n=1, phenobarbital	(24)
2	Witteler *et al*, 2021	Retrospective analysis	3b	n=31, radiotherapy	(18)
3	Wu *et al*, 2021	Systematic review	1a	NA	(36)
4	Harrison *et al*, 2021	Retrospective cohort study	3b	n=132, NA	(25)
5	Lin *et al*, 2020	Retrospective analysis	3b	n=50, medications: Steroids, anti-epileptic drugs, Benzodiazepines….. Allied health involvement: Physiotherapy, occupational therapy, social work, speech pathology, pastoral care	(26)
6	Kim *et al*, 2020	Prospective study	2b	n=294, NA	(35)
7	Golla *et al*, 2020	Randomized clinical trial	1b	n=214, Interventional group [proactive early integration of palliative care (EPIC) on a monthly basis], control group (receiving treatment according to international standards and additional, regular assessment of quality of life)	(37)
8	Glynn *et al*, 2019	Retrospective analysis	3b	n=104 radiotherapy (hypofractionated palliative radiotherapy)	(27)
9	Stavrinou e *et al*, 2018	Retrospective analysis	3b	n=259, glioblastoma was treated with maximal safe resection followed by adjuvant radiotherapy + post-operative chemotherapy (6 cycles of temozolomide), then palliative care with supportive treatment	(14)
10	Hemminger *et al*, 2017	Retrospective analysis	3b	n=117, chemotherapy	(28)
11	Kuchinad *et al*, 2017	Retrospective analysis	3b	n=100, chemotherapy	(29)
12	Thier *et al*, 2016	Prospective study	2b	n=57, non-steroidal anti-inflammatory drugs, anticonvulsants and steroids	(16)
13	Check *et al*, 2014	Case report	4	n=1, mifepristone	(19)
14	Sundararajan *et al*, 2014	Retrospective cohort study	3b	n=678, palliative care consult, palliative care bed, social work, physiotherapy, occupational therapy, speech pathology, psychology, rehabilitation bed	(30)
15	Pompili *et al*, 2014	Prospective study	2b	n=197, 122 of which with GBM, sedation with midazolam, intramuscular phenobarbital for seizure, hydration, tube feeding	(34)
16	Walbert and Khan, 2014	Systematic literature review	1a	NA, interventions include hydration, urinary catheterization, steroids, antiepileptic drugs, oxygen insufflation, tube feeding and palliative sedation	(17)
17	Ziobro *et al*, 2008	Retrospective analysis	3b	n=5,124 palliative treatment with temozolomide	(31)
18	Oberndorfer *et al*, 2008	Retrospective analysis	3b	n=29, the majority of patients were on antiepileptic drugs (AEDs), steroids, and analgesics.	(32)
19	Reimer *et al*, 1998	Prospective study	2b	n=4, laser-induced thermotherapy	(33)

Study type and level of evidence were categorized using the Oxford Centre for Evidence-Based Medicine (CEBM) Levels of Evidence.Levels range from 1a (systematic reviews of RCTs) to 4 (case series and case reports): 1a, systematic review of RCTs; 1b, individual RCT; 2b, prospective cohort study; 3b, retrospective cohort or case-control study; 4, case report or case series study. NA, not available in the study; GBM, glioblastoma.

**Table II:** *G*eographical distribution of the included studies*.*

Article no.	Authors: Study title	Country	(Refs.)
1	Senderovich *et al*: Evading Seizures: Phenobarbital Reintroduced as a Multifunctional Approach to End-of-Life Care.	Published online (Author nationalities: Canada, Ireland, Anguilla)	(24)
2	Witteler *et al*: Palliative radiotherapy of primary glioblastoma.	Germany	(18)
3	Stavrinou *et al*: Survival effects of a strategy favoring second-line multimodal treatment compared to supportive care in glioblastoma patients at first progression.	Germany	(14)
4	Hemminger *et al*: Palliative and end-of-life care in glioblastoma: Defining and measuring opportunities to improve care.	USA	(28)
5	Kuchinad *et al*: End of life care for glioblastoma patients at a large academic cancer center.	USA	(29)
6	Thier *et al*: The Last 10 Days of Patients With Glioblastoma: Assessment of Clinical Signs and Symptoms as well as Treatment.	Austria	(16)
7	Check *et al*: Evidence that mifepristone, a progesterone receptor antagonist, can cross the blood brain barrier and provide palliative benefits for glioblastoma multiforme grade IV.	USA	(19)
8	Sundararajan *et al*: Mapping the patterns of care, the receipt of palliative care and the site of death for patients with malignant glioma.	Australia	(30)
9	Lin *et al*: Inpatient palliative care consultation for patients with glioblastoma in a tertiary hospital.	Australia	(26)
10	Ziobro *et al*: Effects of palliative treatment with temozolomide in patients with high-grade gliomas.	Poland	(31)
11	Oberndorfer *et al*: The end-of-life hospital setting in patients with glioblastoma.	Austria	(32)
12	Reimer *et al*: MR-monitored LITT as a palliative concept in patients with high grade gliomas: Preliminary clinical experience.	Germany	(33)
13	Wu *et al*: Palliative Care Service Utilization and Advance Care Planning for Adult Glioblastoma Patients: A Systematic Review.	USA	(36)
14	Kim *et al*: Utilizing a Palliative Care Screening Tool in Patients With Glioblastoma.	USA	(35)
15	Golla *et al*: Effect of early palliative care for patients with glioblastoma (EPCOG): a randomised phase III clinical trial protocol.	Germany	(37)
16	Pompili *et al*: Home palliative care and end of life issues in glioblastoma multiforme: results and comments from a homogeneous cohort of patients.	Italy	(34)
17	Walbert and Khan: End-of-life symptoms and care in patients with primary malignant brain tumors: A systematic literature review.	USA	(17)
18	Glynn *et al*: Glioblastoma Multiforme in the over 70's: ‘To treat or not to treat with radiotherapy?’.	Ireland	(27)
19	Harrison *et al*: Aggressiveness of care at end of life in patients with high-grade glioma.	USA	(25)

**Table III:** Summary of the included studies.

First author	Aim of study	Total no. of patients	GBM diagnosis duration	Patients KPS score or ECOG score (if mentioned)	Treatment of the GBM (surgery, radiation, chemotherapy, no treatment or others)	Palliative care treatment type	PC inpatient vs. outpatient	Palliative care treatment	Study outcomes	Median survival rate of patients	Recommendations	(Refs.)
Pompili	Identify home palliative care and end of life issues in GBM	197: Brain tumors, 122 of them GBM	NM	KPS score >70	NM	Supportive treatment	Outpatient	Sedation with midazolam + Intramuscular phenobarbital for seizure + hydration + tube feeding	1-End of life palliative sedation with midazolam was necessary in 11% of cases to obtain good control of symptoms such as uncontrolled delirium, agitation, death rattle, or refractory seizures. 2-Intramuscular phenobarbital is the authors' drug of choice for the severe seizures that occurred in 30% of cases.	13.34 months	1-Future clinical research strategies should include new models of care for patients with brain tumors, with special attention given to palliative home care models.	(34)
Oberndorfer	To evaluate the end-of-life phase in a hospital setting in patients with GBM.	29	NM	Mean KPS phase 1= 70%/Phase 2= 50%/Phase 3= 20%	Surgery + radiotherapy + subsequent chemotherapy/7-Surgery only (n=4), or biopsy (n=3)	Symptoms drug + physiotherapy + occupational therapy + logopedia + psychologic assessment-directed to mobilize the patient and to strengthen his remaining function, was only marginal in all three phases	Inpatient/outpatient	Antiepileptic drugs (AED) + steroids + analgesics	1-End of life in patients with glioblastoma has several periods with different clinical aspects with respect to symptoms and treatment/2-Drug treatment generally showed a continuous increase from phase 1 to 3 except steroids, which declined in phase 3.	The last 10 weeks before death were divided into three periods. Phase 1, from 10 to 6 weeks before death; phase 2, 6 to 2 weeks before death; and phase 3, the last 2 weeks before death	1-In Phase 3-All medication should be promptly available and possibly given by a nonoral route because dysphagia is present in the majority of patients. 2-Practice of sedation in terminal ill patients has a wide divergence among palliative care specialists and no clear guidelines are available. 3-The requirement of further clinical research to develop evidence-based guidelines.	(32)
Kuchinad	Retrospectively analyze end-of-life care for GBM patients at academic center and compare utilization of these services to national quality of care guide, lines, Identifying opportunities to improve end-of-life care.	100	NM	NM	Chemotherapy	NM	Inpatient/outpatient	chemotherapy	1-Documentation of palliative care and end-of-life measures could improve quality of care for GBM patients, especially in the use of ADs, symptom, spiritual, and psychosocial assessments, with earlier use of hospice to prevent end-of-life hospitalizations. 2-Hospice referral and enrollment at Johns Hopkins exceeded national standards while documentation of advance directives, and psychosocial assessments demonstrated room for improvement.	22 days	1-More research is needed to further define appropriate symptom management and end-of-life care for this population. 2-Collaboration amongst providers including neuro-oncologists, medical oncologists, radiation oncologists, neuro-surgeons, social workers, chaplains and other members of the care team can help optimize utilization of palliative care measures at the end-of-life and identify and establish necessary palliative care measures specific to the GBM population.	(29)
Walbert	Review the literature on end-of-life symptoms and end-of-life care of adult patients with high-grade glioma (HGG).	NA	NM	NM	NM	Palliative care interventions	Inpatients =3 studies/outpatients =2 studies/both =2 studies	Hydration + urinary catheterization+ steroids + antiepileptic drugs + oxygen insufflation + tube feeding + palliative sedation	1-Patients with HGG have a significant symptom load that worsens markedly at the end of their lives (Poor communication, speech impairments, and cognitive decline are common at the end-of-life period).	NM	1-More prospective studies are needed to better understand the end-of-life phase of brain tumor patients. 2-Interventions should be evaluated to reduce symptom burden and improve quality of life for patients and carers without compromising the hope paradigm.	(17)
Reimer	Evaluate the clinical utility of laser-induced thermotherapy (LITT] as a palliative treatment for patients with high-grade glioma	4	NM	NM	Surgery + radiotherapy	Laser-induced thermotherapy	Inpatient	Laser-induced thermotherapy	1-Interventional MRI-controlled LITT offers a number of potential treatment benefits/2-MRI provides excellent topographic accuracy because of its capability for soft tissue contrast, high spatial resolution, and functional aspects.	NM	1-The results have yet to be verified in a larger clinical trial and then to be compared with those of various other minimally invasive techniques. 2-Radiofrequency ablation, focused ultrasound, or cryosurgery are alternative methods for tissue coagulation/ablation and have been described for the ablation of brain tumors.	(33)
Lin	Examining the symptoms, reasons for referral and outcomes of patients with GBM referred to inpatient palliative care service.	50	The median time from diagnosis of GBM to the palliative care consultation service referral was 111 days (range 3-1,677).	NM	94%-Surgery/54%-Radiotherapy/48%-chemotherapy	NM	Inpatient	Medication - Steroids + Anti-epileptic drugs + Benzodiazepines/Allied health involvement-Physiotherapy + Occupational therapy + Social work + Speech pathology + Pastoral care	1-Early palliative care review of cancer patients can result in significant improvements in pain, somnolence, and symptom distress scores as well as overall well-being/2-The improvements were observed within the first few days of consultation/3-Allied health services, rehabilitation and psychosocial support are crucial components of patient management.	The median time from referral to date of death was 33 days (range 0-256), The median length of inpatient stay was 9 days (range 2-35). The median time from diagnosis of GBM to the palliative care consultation service referral was 111 days (range 3-1677).	1-Allied health services, rehabilitation and psychosocial support are crucial components of patient management	(26)
Check	Determine if mifepristone could provide palliative benefits to patient with end-stage stage IV glioblastoma multiforme	1	NM	NM	radiation + chemotherapy	*progesterone* *receptor* *antagonist*	NM	*mifepristone*	1-mifepristone cross the blood-brain barrier and could be considered for palliative therapy of other patients with chemotherapy-resistant brain cancer/Within two weeks of taking mifepristone, patient became more alert and able to carry-out intelligent.	NM	1-Mifepristone does cross the blood-brain barrier and could be considered for palliative therapy of other patients with chemotherapy-resistant brain cancer. Further studies are required to determine if the 35-kDa isoform of PIBF described by Lachman et al. in the cytoplasm of cancer cells is identical to the 34-kDa form that rises.	(19)
Hemminger	Evaluate adherence to 5 palliative care quality measures and explore associations with patient outcomes in GBM	117	Diagnosis between January 1, 2010 and May 1, 2015	NM	Chemotherapy	Hospice care	Inpatient= 31/outpatient= 12	NM	Early PC help with: 1-Reduce symptom burden. 2-Decrease rates of depression in patients and caregivers. 3-Reduce costs of care. 4-Minimize hospitalizations and in-hospital deaths. 5-Decrease aggressive end-of-life care. 6-Improve a patient's survival./but the study results are consistent with the literature in illustrating that early involvement of palliative care services is rare in neuro-oncology.	12.9 months	1-Quality measures in glioblastoma should focus on defining early advance directive documentation, suggesting appropriate timing for hospice enrollment, and determining which patients may benefit from early palliative care interventions (GUIDELINES ARE NEEDED).	(28)
Wu	Exploring published literature on the prevalence of ACP, end-of-life (EOL) services utilization (including PC services), and experiences among adults with GBM	NA	Median time from diagnosis to PC consult measured in one study, found to be 111 days	NM	NM	NM	Inpatient/outpatient	NM	1-Proactive advance care planning and appropriate use of palliative care resources are critical aspects of high-quality care for these patients and their caregivers/2-our findings suggest relatively low prevalence of both of these components among GBM patients.	NM	1-The field would benefit from rigorous studies, particularly involving prospective cohorts, to inform future improvements in ACP and EOL care for adult GBM patients as well as to explore other pertinent topics.	(36)
Kim	Investigate the feasibility, value, and effectiveness of using an adapted palliative care screening tool to improve out-patient palliative care screening and referral of glioblastoma patients	294	NM	90-100% 70-80% 50-60% 30-40% 10-20% 133 (45%) 123 (42%), 35 (12%), 3 (1%)	NM	NM	Outpatient	NM	1-Utilizing a palliative care screening tool may facilitate early referral to palliative care and lead to improved patient outcomes in symptom management and quality of life.	NM	1-In future studies, a query about patient acceptance regarding palliative care is required to identify the most effective and efficient model of early palliative care integrated with oncology care.	(35)
Sundararajan	Quantify the association between symptoms, receipt of supportive and palliative care and site of death.	678	NM	NM	NM	Palliative care consult + Palliative care bed + Social work + Occupational therapy + Physiotherapy	inpatient	Palliative care consult + Palliative care bed + Social work + Physiotherapy + Occupational therapy + Speech pathology + Psychology + Rehabilitation bed	Malignant glioma patients with a high burden of symptoms more likely to receive palliative care/Patients who receive palliative care more likely to die at home	10.4 months, and 14.3 months for all other patients with grade three tumors. 821 (41%) did not die by the end of follow up (30-June-2009), leaving 678 (34%) patients who survived longer than 120 days from diagnosis and died within the follow-up period.	1-Model of care for this population should incorporate an earlier routine palliative care referral, heralded by the onset of symptoms. The response of treating clinicians to a relapse may include further anti-cancer therapies, but should also routinely offer referral to palliative care. For patients whose survival may be measured in months, this should ensure receipt of palliative care involvement prior to their last days of life.	(30)
Senderovich	Evaluate the role of phenobarbital as a drug of choice in end-of-life (EOL) settings.	1	NM	NM	NM	Subcutaneous drug	NM	Phenobarbital	1-Phenobarbital reduced complications associated with EOL care + improve quality of remaining life.	NM	1-Information regarding phenobarbital use for EOL care is underwhelming and clearly should be further explored.	(24)
Witteler	Identify predictors of survival after palliative radiotherapy	31	NM/select patients diagnosed with GBM between (2006-2019)	Patients with (KPS >= 60) showed improved survival compared to those with (KPS=<50)	Surgery (Subtotal resection or biopsy) + radiotherapy	Radiotherapy	inpatient	radiotherapy	1-Palliative radiotherapy increase in survival + reasonable option for patients with limited survival prognoses.	NM	1-Results need to be confirmed in a larger prospective trial.	(18)
Glynn	Analyze survival data and determine predictors of survival in patients aged ≥70 years treated with radiotherapy (RT) and/or Temozolomide.	104	NM	NM	Radiotherapy +/- chemotherapy	Radiotherapy	NM	Radiotherapy (hypofractionated palliative RT)	1-Patients aged 70-75 years had survival rates similar to younger age groups. 2-Patients undergoing palliative RT had worse results. 3-Increasing age was associated with poorer outcomes and decreased survival. 4-Age, surgical debulking, and good performance status were independent predictors of improved survival.	6.0 months	1-Maximal surgical resection if feasible for all ages. 2-For patients aged 70-75 years, if they have Debulked and good performance status, standard approach radical RT/TMZ, 3-If they have biopsy only and good performance status they recommend a standard approach radical RT/TMZ versus short course RT (±TMZ), 4-If they have Poor performance status they reccomaned to discuss short course RT (±TMZ) versus best supportive care (BSC). 5-for patient aged more than 76 years and they have good performance status and Debulked they recommended to discuss short course RT (±TMZ) versus BSC, 6-If they have Biopsy only and poor performance status they reccomanded to have BSC.	(27)
Stavrinou	Examine whether a strategy favoring active treatment of GBM at progression offers an advantage in OS compared to supportive care alone.	259 (center A=103/center B=156)	June 2010-June 2015	Center A= 91/center B=146	Surgery + adjuvant radiotherapy + postoporative chemotherapy (6 cycles of temozolomide)	Supportive care	NM	supportive	1-Treatment favoring second-line treatment GBM recurrence or progression is associated with significantly better survival after progression.	Center A= 4.5 months/Center B= 7 months	NM	(14)
Ziobro	Assess the results of treatment with temozolomide in patients with high-grade gliomas who no longer benefit from surgical treatment and radiotherapy.	51,24	NM	NM	Surgery + Radiotherapy + Chemotherapy (lomustine)	Speech pathology + Psychology + Pharmacy + Rehabilitation bed	NM	Temozolomide	1-Objective benefit from treatment with temozolomide was noted in 49% of patients in the study group/2-Tolerability of temozolomide in patients with malignant gliomas is good.	32 weeks	NM	(31)

GBM, glioblastoma; KPS, Karnofsky performance status; NM, not mentionedin the study.

**Table IV:** Location of mortality reported in the included studies.

Location of mortality	Percentage of mortality	No. of studies
Home	33.33	7
Hospital	57.14	10
Hospice	19.05	4
NM	42.86	9

The different locations of the mortality of patients with glioblastoma receiving palliative care. The hospital was the most commonly reported place of mortality (57.14%), while the least reported place of mortality was a hospice (19.05%). A total of 7 studies (33.33%) reported that their patients succumbed in their own home. However, 9 (47.86%) studies did not mention the location of mortality of their patients. NM, not mentioned.

## Data Availability

The data generated in the present study may be requested from the corresponding author.
